# Frequent lineage-specific substitution rate changes support an episodic model for protein evolution

**DOI:** 10.1093/g3journal/jkab333

**Published:** 2021-09-20

**Authors:** Neel Prabh, Diethard Tautz

**Affiliations:** f Department of Evolutionary Genetics, Max Planck Institute for Evolutionary Biology, August-Thienemann-Str. 2, 24306 Plön, Germany; 1 Department of Evolutionary Genetics, Max Planck Institute for Evolutionary Biology, Plön 24306, Germany

**Keywords:** episodic evolution, molecular clock, divergence, lineage-specific, synteny, ortholog

## Abstract

Since the inception of the molecular clock model for sequence evolution, the investigation of protein divergence has revolved around the question of a more or less constant change of amino acid sequences, with specific overall rates for each family. Although anomalies in clock-like divergence are well known, the assumption of a constant decay rate for a given protein family is usually taken as the null model for protein evolution. However, systematic tests of this null model at a genome-wide scale have lagged behind, despite the databases’ enormous growth. We focus here on divergence rate comparisons between very closely related lineages since this allows clear orthology assignments by synteny and reliable alignments, which are crucial for determining substitution rate changes. We generated a high-confidence dataset of syntenic orthologs from four ape species, including humans. We find that despite the appearance of an overall clock-like substitution pattern, several hundred protein families show lineage-specific acceleration and deceleration in divergence rates, or combinations of both in different lineages. Hence, our analysis uncovers a rather dynamic history of substitution rate changes, even between these closely related lineages, implying that one should expect that a large fraction of proteins will have had a history of episodic rate changes in deeper phylogenies. Furthermore, each of the lineages has a separate set of particularly fast diverging proteins. The genes with the highest percentage of branch-specific substitutions are ADCYAP1 in the human lineage (9.7%), CALU in chimpanzees (7.1%), SLC39A14 in the internal branch leading to humans and chimpanzees (4.1%), RNF128 in gorillas (9%), and S100Z in gibbons (15.2%). The mutational pattern in ADCYAP1 suggests a biased mutation process, possibly through asymmetric gene conversion effects. We conclude that a null model of constant change can be problematic for predicting the evolutionary trajectories of individual proteins.

## Introduction

The idea of a constant divergence of proteins over time has existed since the initial investigations into protein divergence, which started with examining serological evidence followed by the analysis of hemoglobin homologs (Nuttall 1904; Zuckerkandl and Pauling 1962). Refinement of this idea then led to the formulation of the molecular clock hypothesis of a more or less constant decay of sequence information in genes over evolutionary time (Zuckerkandl and Pauling 1965; Ota and Kimura 1971; Langley and Fitch 1974; Takahata 2007). However, examples that violated the molecular clock pattern were also identified early on, initially in hemoglobin itself (Goodman *et al.* 1975). Based on an extended sampling, Goodman *et al.* noted “. in contradistinction to conclusions on the constancy of evolutionary rates, the hemoglobin genes evolved at markedly nonconstant rates,” pointing out that phases of adaptation can lead to a lineage-specific change of substitution rates. It has generally been observed that the variance of rates in different lineages is often higher than their mean for the given protein families, a phenomenon called overdispersion of the clock, which suggests that the rates are driven by more complex processes than originally assumed (Cutler 2000; Wilke 2004; de la Paz *et al.* 2020). Also, genome-wide studies on protein families in given taxon groups have suggested lineage-specific acceleration and deceleration patterns for a subset of protein families (Jordan *et al.* 2001; Kawahara and Imanishi 2007; Shapiro and Alm 2009; Toll-Riera *et al.* 2011). Still, in cumulative studies across many genes, the molecular clock pattern is often supported and is systematically used to compile divergence times for the tree of life (Kumar *et al.* 2017).

The question of rate constancy versus lineage-specific acceleration or deceleration has acquired new relevance in the context of understanding the evolution of orphan genes. For about a third of proteins in a given genome, one cannot find homologs in distant lineages (Khalturin *et al.* 2009; Tautz and Domazet-Lošo 2011). It was initially assumed that these so-called orphans have evolved through fast divergence after a duplication event has occurred. But systematic tests of evolutionary rates of orphans have shown that at least some of them show very low evolutionary rates that are comparable to highly conserved and universally detectable genes. This has raised the possibility that such proteins have undergone an episodic phase of fast evolution after their duplication, until they assumed a new functional role that resulted in the more constrained rate that is observed in the respective extant lineages (Domazet-Loso and Tautz 2003). But nowadays, it is often assumed that orphan genes are also derived through *de novo* evolution from noncoding sequences (Tautz and Domazet-Lošo 2011; Prabh and Rödelsperger 2019; Van Oss and Carvunis 2019), *i.e.*, would not necessarily have had an episodic history of rate changes. Under this assumption, Weisman *et al.* (2020) have recently proposed to use constant family-specific decay rates as a null hypothesis for judging whether a given protein family diverges simply by constant decay into orphan status, or whether it could be a candidate for *de novo* gene emergence (Weisman *et al.* 2020). However, the application of such a procedure could be problematic if many protein families do not adhere to a constant decay rate over time.

Due to the fast increase of genomic data from a broad range of taxa, one could expect that systematic estimates of protein decay rates to resolve this question should be straightforward. However, it remains a nontrivial problem due to three main reasons. First, separating orthologs from paralogs is not straightforward, and it gets further complicated as one moves deeper into the phylogeny. Alignment of paralogs can create a systematic problem in divergence rate estimation (Forslund *et al.* 2018; Glover *et al.* 2019). Second, insertion and deletion within genes make alignment and recognition of substitution events less reliable (Ebersberger and von Haeseler; Cantarel *et al.* 2006; Talavera and Castresana 2007; Lunter *et al.* 2008; Wong *et al.* 2008; Markova-Raina and Petrov 2011). Third, one cannot automatically scale model-based evolutionary rate estimation methods, such as dN/dS analysis to the genome level, mainly because their underlying parameters are independently calculated for each gene family. Also, these methods assume that the dS evolves under neutral rates, but this assumption has been challenged (Chamary *et al.* 2006; Parmley and Hurst 2007; Hurst 2009, 2011; Plotkin and Kudla 2011; Wang *et al.* 2011). To avoid the confounding problems around noncoding substitution rates, we focus our study on the original approach of estimating decay rates, *i.e.*, on direct amino acid sequence comparisons.

With the availability of large datasets, alignments of protein sequences became automatized to handle such comparisons efficiently while accepting that this creates noise in the case of suboptimal alignments around indels or highly diverged regions (Ebersberger and von Haeseler; Cantarel *et al.* 2006; Talavera and Castresana 2007; Lunter *et al.* 2008; Wong *et al.* 2008; Markova-Raina and Petrov 2011). Hence, getting reliable data for divergence rates requires alignment optimization. Furthermore, whole-genome data have shown that misalignments between duplicated copies of the genes can be a major impediment and need to be systematically addressed (Forslund *et al.* 2018; Glover *et al.* 2019).

We have produced here a highly curated dataset of four species from the ape phylogeny, including humans, to revisit the decay rate constancy question for a large part of these genomes’ known coding sequences. We identify hundreds of lineage-specific slow and fast diverging proteins and other proteins with complex evolutionary trajectories. We conclude that there is a high probability of acceleration and deceleration of substitution rates for many genes, even at short evolutionary time scales. Projecting this to larger evolutionary time scales, one should expect that a large fraction of protein families should have been subject to lineage-specific substitution rate changes at some point in their history. Such fluctuations may for a given protein result in bursts of rapid acceleration followed by periods of strong conservation that may cancel each other. Although this can yield a long-term constant rate pattern, the actual history of protein sequence evolution can be much more complex. Hence, we conclude that the classic alternative to a null model of constant decay, namely episodic evolution (Hudson 1983; Gillespie 1984), is the more appropriate model for understanding protein family evolution.

## Methods

### One to one orthologs

For each species, we downloaded the CDS fasta file and gff file from the Ensembl ftp server (release-98) (Yates *et al.* 2020). We extracted the fasta sequence for the CDS of each gene’s longest isoform categorized as “biotype: protein-coding” in the gff file for further analysis. We translated the extracted CDS fasta sequences to obtain their corresponding protein sequence. To detect homologous genes for each pair of species in our analysis, we ran all vs all BLASTP (Albà and Castresana 2007). The BLASTP result file and gff files of each species pair were provided as an input to MCScanX for synteny ascertainment (Wang *et al.* 2012). MCScanX was allowed to call a collinear block if a minimum of three collinear genes were found for the species pair with a maximum gap of two genes in between (Figure 2A). Several recent studies also relied on collinearity to establish orthologous relationships (Heger and Ponting 2007; Lu *et al.* 2017; Rödelsperger *et al.* 2017; Sieriebriennikov *et al.* 2018; Zhang *et al.* 2019; Zhao and Schranz 2019; Vakirlis *et al.* 2020b).

We parsed the collinear gene pairs obtained from the MCScanX using the following method:

1. Both protein sequences from each collinear gene pair were aligned with Stretcher (Madeira *et al.* 2019).
2. If both proteins had 95% or more sequence identity, then this syntenic gene pair was retained.
  - Else, we checked if either gene has a better BLASTP match, based on the BLASTP bit score, with another gene from the other species. If so, we removed the gene pair.
3. If a gene was present in more than one syntenic pair, we retained the pair within the larger syntenic block (based on the number of genes within each block).
4. Gene pairs with either gene identified as a tandem duplicate by MCScanX were removed.

**Figure 1:**
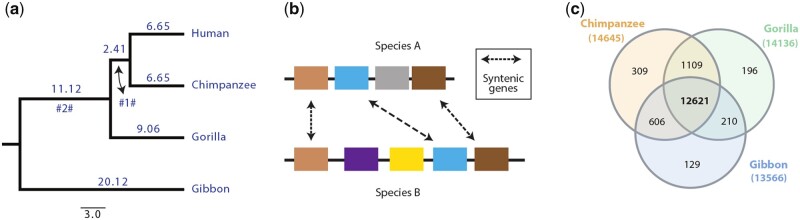
Syntenic orthologs. (A) Phylogeny of apes adapted from www.timetree.org (Kumar *et al.* 2017), branch length is divergence time in mya. #1# and #2# are the two internal branches. (B) Same color boxes represent the collinear or syntenic orthologs between the two species A and B.(C) Venn diagram depicting the overlap of human genes syntenic with the other three apes.

Thus, in the end, we were left with a list of 1:1 orthologs for the given species pair (Figure 1C).

We overlapped the list of 1:1 orthologs of human genes with chimpanzee, gorilla, and gibbon genes to determine the ortholog gene families. We retained only those gene families that have orthologs of the human genes in all three lists (Figure 1B). The length variation within the ortholog families was calculated by subtracting the shortest ortholog’s length from the longest ortholog.

### Multiple sequence alignment of orthologous gene families

To investigate protein sequence divergence caused by single nucleotide substitution, we need to align amino acid residues that are derived from the same site of their last common ancestor. Given that most gene families are of comparable length, we set out to create alignments with fewer gaps. Hence, MAFFT was run with a gap opening penalty of 3 (Katoh and Standley 2013). Then to remove unreliable columns from the alignment, we used Gblocks with the following parameters (Talavera and Castresana 2007):

1. Minimum Number of Sequences for A Conserved Position:4
2. Minimum Number of Sequences for A Flanking Position:4
3. Maximum Number of Contiguous Nonconserved Positions:2
4. Minimum Length of A Block:10
5. Allowed Gap Positions:0

Thus, in the end, we were left with the concatenation of all the conserved blocks identified by Gblocks. These blocks were free of any gap and at most contained two substituted sites contiguously. Given the phylogenetic proximity of all four species under investigation, we assumed that it was unlikely that many instances of three or more contiguous amino acid substitutions would result from independent point substitution events. Therefore, to avoid the inclusion of insertion or deletion events within the alignment block, we have removed any gaps or contiguous substitution of three and more residues.

Since gaps are not allowed in our alignment, their maximum length is limited by the shortest ortholog. We use this qualification to measure the completeness of every alignment. If the overall alignment length is equal to the shortest ortholog’s length, this family will have attained 100% alignment saturation. The ‘Alignment Saturation’ level is calculated as per the following formula:

$$\mathrm{Alignment}\;\mathrm{Saturation}\;=\;\frac{\mathrm{Alignment}\;\mathrm{overlap}}{\mathrm{Length}\;\mathrm{of}\;\mathrm{the}\;\mathrm{shortest}\;\mathrm{sequence}\;}\;\times 100\%$$

Alignmentoverlap=Number of aligned sites

### Relative Branch length and % substitutions per site

The relative branch lengths were calculated as per the following formula:

$$\mathrm{Relative}\;\mathrm{length}\;\mathrm{of}\;\mathrm{Branch}\;A\;=\;\frac{\mathrm{Total}\;\mathrm{Substitutions}\;\mathrm{on}\;\mathrm{Branch}\;A}{\mathrm{Total}\;\mathrm{Branch}-\mathrm{specific}\;\mathrm{substitutions}}$$

The % substitutions per site were calculated as per the following formula:

$$\%\;\mathrm{substitutions}\;\mathrm{per}\;\mathrm{site}\;=\;\frac{\mathrm{Number}\;\mathrm{of}\;\mathrm{substituted}\;\mathrm{sites}}{\mathrm{Alignment}\;\mathrm{overlap}\;}\;\times 100\%$$

For total % substitutions per site, all substituted sites were used in the above formula. But for branch-specific substitution frequencies, only branch-specific sites were used. The branch-specific sites were identified as sites with species-specific substitution *i.e.*, only one substitution in any given column that is specific to one of the four species sequences. #1#-specific substitutions were identified as columns with human-chimpanzee identity and gorilla-gibbon identity, as shown in Figure 3A.

We obtained the expected number of “No identity” sites based on the following assumptions and calculations:

1. A “No identity” site must undergo at least three independent substitution events.
2. The three lineages with the highest substitution rates in our analysis were gibbon, gorilla, and #1# plus human, with 0.0134, 0.0036, and 0.0036 substitutions per site, respectively.
3. Given that there were 7,313,620 aligned sites, the expected number of sites substituted on all the above three lineages is:
  Expected ‘No identity’ sites = 0.0134 ×0.0036 ×0.0036 × 7313620 =1.27

### Poisson corrected (PC) branch length

The PC length for each branch was calculated using the following formula:

$$\mathrm{PC}\;\mathrm{length}\;\mathrm{of}\;\mathrm{Branch}\;A\;=\;-\ln\;(1-\;\frac{\mathrm{Substitutions}\;\mathrm{on}\;\mathrm{Branch}\;A}{\mathrm{Alignment}\;\mathrm{overlap}})$$

### RF metric and tree comparison

We computed the mean tree for the substitute families by obtaining the average PC length for each branch (Table 1). For every family, first, the “RF branch-score” for each branch was calculated as the absolute difference (only the value of the difference, not its sign) between the PC length for the given branch of the family and the mean tree. Then the RF score for the family was obtained by adding all RF branch-scores using the following formula:

$$\text{RF score}=\;\overset{\text{All branches}}{\underset{\text{b}=\text{A}}{\sum }}|{\text{ PC}}_{\text{b}}-\;{\underline{\text{PC}}}_{\text{b}}|$$

**Table 1:** Branch-specific substitution rates

	Branch timetree (Mya)	Total subs	Relative branch length	% subs per site	% subs per site per Mya from average	Mean tree PC length
Human	6.65	19815	0.1174	0.27	0.041	0.003
Chimpanzee	6.65	18818	0.1115	0.26	0.039	0.003
#1#	2.41	6279	0.0372	0.09	0.036	0.001
Gorilla	9.06	26026	0.1542	0.36	0.039	0.004
Gibbon	31.24	97830	0.5797	1.34	0.043	0.015

Here, ${PC}_{b}$ is the Poisson corrected length of branch A for the given family and ${\underline{PC}}_{b}$ is the average Poisson corrected length of branch A of the mean tree (Table 1). A low RF score indicated that the family tree was close to the mean tree and thus diverged at a similar rate. We conducted a standard Z-test to evaluate if the RF score for the given family was significantly different from zero. The variance of RF score V$(z_{i}$) was estimated from 1000 bootstrap replications for the entire underlying alignment by using the following formula (Efron 1982; Efron and Tibshirani 1994; Nei and Kumar 2000; Kumar and Filipski 2001):

$$V(z_{i})=\frac{1}{\left(B-1\right)}{\sum_{b=1}^{B}}_{}^{}{\left(z_{i}b-z_{i}\right)}^{2}$$

Here, *B *=* *1000 (number of bootstrap replicates), $z_{ib}$ is the value of $z_{i}$ estimated at the *b*^th^ bootstrap replication, and ${\underline{z}}_{i}$ is the average of the $z_{ib}$.

We applied the FDR (false discovery rate) method for multiple testing corrections. The statistical significance of RF scores were used to draw stacked histograms for the PC tree length distribution (Figure 4). For each family, the PC tree length was computed by adding all the branches of the given tree.

### Branch-specific fluctuation from the expected rate

We extracted a list of total branch-specific substitution events “N” observed in every ortholog family. For every value of N observed in our data, we performed 100,000 simulations where the probability of a substitution event falling on a given branch was equal to its relative branch length (Table 1). The simulation runs provided a null distribution of substitutions on each branch for ortholog families with overall N branch-specific substitutions (see *supplementary material*). Based on this distribution, we performed for each ortholog family with the respective number of substitutions a two-tailed rank test to obtain the *P*-value of finding the observed or more extreme value of substitutions on the given branch. This was repeated for all five branches of the given ortholog family. Bonferroni correction for all five branches being tested in each family resulted in an adjusted *P*-value threshold of 0.01, which was used to detect all significant deviations from the expected rate.

### Identification and analysis of most divergent genes

The top five candidates on each hominid branch, from the candidates already identified to have higher than expected substitutions on the given branch, were manually curated after sorting to their % substitutions per site. Further, we visually inspected the alignments and removed candidates that were not fully reliable. Such filtered candidates are flagged in the “Comment” section of the “NormDf.tsv” file. The top 5 human candidates were validated with tissue-specific human expression data and Ensembl CDS alignment (Fagerberg *et al.* 2014). The duplicates of candidate genes were identified based on the Ensembl database’s paralogue information (Yates *et al.* 2020). The nucleotide alignment of ADCYAP1 was manually created.

### Statistical analysis

Statistical analysis and tests were done using custom python codes. All default uncorrected *P*-value thresholds were at 0.05.

## Results

### Synteny guided ortholog identification

To study lineage-specific divergence at the amino acid residues level, we started with an identification of orthologous proteins in the extant species. To ascertain this, we chose four ape species: human, chimpanzee, gorilla, and gibbon (Figure 1A). They have a well-documented evolutionary history, and their overall genome divergence is sufficiently small to ensure unambiguous alignments of proteins. Furthermore, the human genome is among the best-curated genomes and serves as a reliable reference for comparisons. We identified the orthologs of the genes annotated in humans by combining reciprocal best BLAST hits and the pairwise analysis of gene order (Figure 1B). Among the 19,976 annotated human genes in the study, we found 14,645 syntenic with chimpanzee, 14,136 with gorilla, and 13,566 with white-cheeked gibbon (Figure 1C). Note that there are large-scale chromosomal rearrangements in the gibbon genome, but at the smaller-scale, it is largely comparable with the other apes (Carbone *et al.* 2014). In total, we retrieved 12,621 ortholog gene families shared between the four species, which represents about two-thirds of the annotated human genes. The failure to identify definite orthologs for the remainder of the genes is mostly due to duplication patterns that could not be fully resolved based on our strict filtering criteria (see Methods). Still, this constitutes the largest gene set comparison analyzed for these species so far.

### Optimized alignment

**Figure 2:**
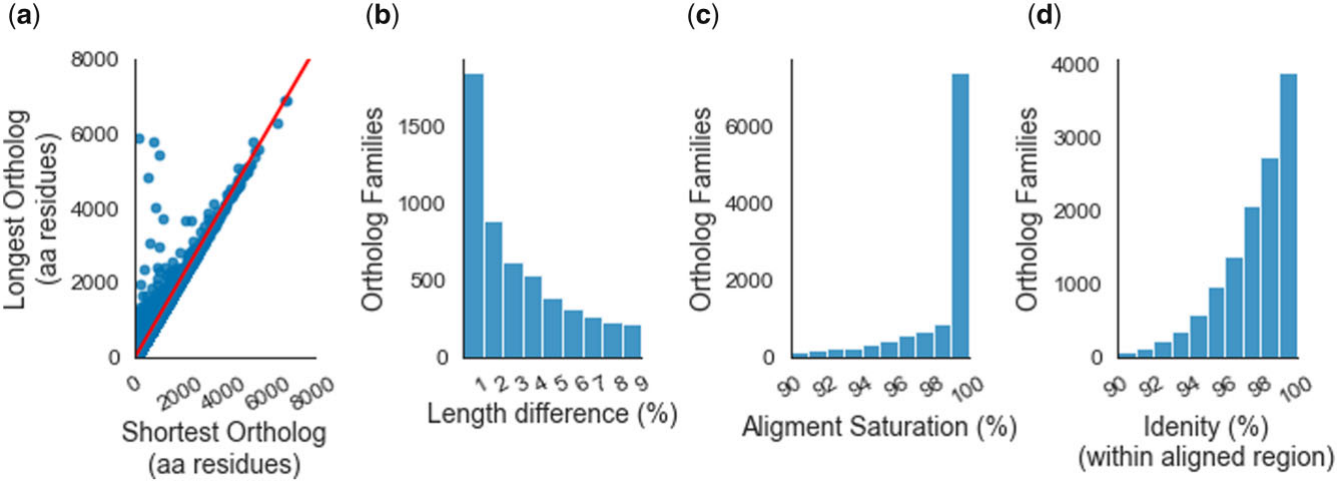
Ortholog families. (A) Scatter plot of the shortest and the longest orthologs of each ortholog family. The regression line is drawn in red. (B) Histogram showing ortholog family distribution for maximum length difference per 100 residues of the shortest ortholog. (C) Histogram showing ortholog family distribution for alignment saturation. (D) Histogram showing ortholog family distribution for identical sites per 100 aligned sites. Lower limits were excluded from the bins in panels B–D.

Proteins can diverge due to amino acid substitutions and changes in the reading frames’ length, either due to new start/stop codons or inclusion/exclusion of exons. Therefore, we have analyzed how far these latter factors influence our gene set by examining the length variation between the longest and shortest orthologs from each family (Figure 2A). One-third of the ortholog families (*N* = 4238) had no length variation, with each ortholog having the same length, another one-third (*N* = 4289) had the longest orthologs that were less than 5% longer than the shortest orthologs (Figure 2B). Thus, confirming that most ortholog families in our analysis were made of proteins that do not show considerable variation in their lengths.

The orthologs’ overall length similarity allowed us to employ stringent criteria (zero gap tolerance and low contiguous substitution threshold) for creating multiple sequence alignments from these families. Only three ortholog families did not overlap in the final alignment due to truncation (see Supplementary File S1); we removed these from further analysis. Thirty-five ortholog families shared less than 50 residue overlap, but we retained these. The presence of nonoverlapping families suggested that our alignment protocol’s rigor could have led to the filtering of a large number of sites. So, we checked whether or not the alignments stretched across the entire length of the shortest ortholog. We estimated our alignments’ completeness by calculating the alignment saturation level, representing the fraction of the smallest ortholog retained in the final alignment. Our results show that nearly half of the ortholog families (*N* = 5805) had 100% alignment saturation, and only 11% of all ortholog families had less than 90% alignment saturation (Figure 2C). The mean saturation level stood at 96.5%. The observation that further bolstered the confidence in our alignments’ quality was that 88% of all ortholog families shared over 95% identity with all four species within the aligned region (Figure 2D).

### Substitution patterns

Of the aligned 7,313,620 amino acid residues in the 12,618 ortholog gene families under investigation, 97.5% were identical in all four species. This leaves 175,284 residues with at least one substitution, of which 162,489 were species-specific substitutions, *i.e.*, they were substituted in only one of the four species, 97,808 of them in gibbons. Note that because no outgroup was used, the internal branch #2# (shown in Figure 1A) was added to the Gibbon branch.

**Figure 3:**
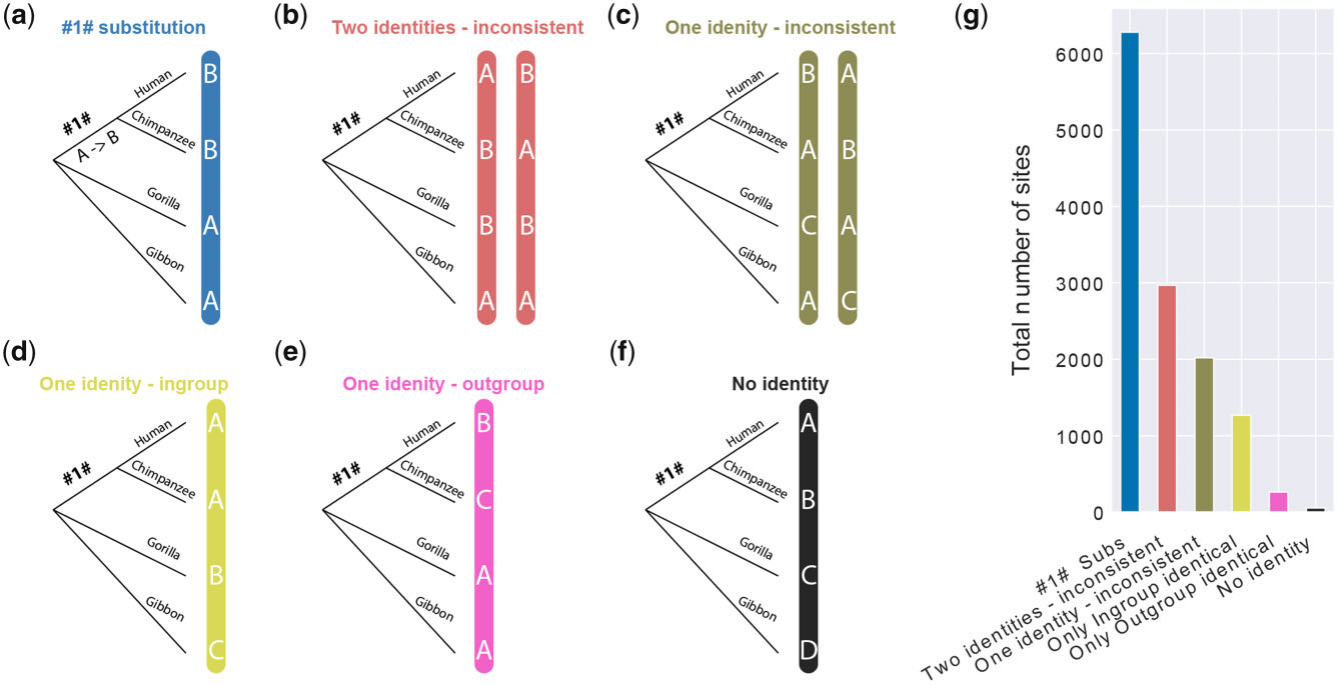
Sites substituted in more than one species. (A–F) Different types of substitution patterns identified from the alignments. Letters in vertical blue bars represent residues on an aligned site. The outgroup Gibbon branch includes the #2# branch from Figure 1A. (G) Bar plot representing the total number of sites based on substitution types.

Twelve thousand, seven hundred, and ninety-five residues in 5407 protein families were substituted in more than one species and are therefore potentially phylogenetically informative. We divided them into six categories (Figure 3, A–F) based on the phylogeny. Interestingly, only about half of these residues with two states (*N* = 6,279) were consistent with the phylogeny (Figure 3, A and G), while 4987 residues with two or three states were phylogenetically inconsistent in different combinations (Figure 3, B and C). Since convergent substitutions are unlikely in this dataset, this testifies the influence of incomplete lineage sorting or introgression effects in this shallow phylogeny. As these can potentially lead to errors in rate estimation (Mendes and Hahn 2016), we removed these residues from further analysis. The remaining residues constitute three or more states (Figure 3, D–F), including the category “No identity,” which covered 52 residues with a different amino acid in each branch (Figure 3F), and each residue was in a different ortholog family. We conjecture that these are hypermutable residues since the estimated expected number of residues with substitution on all four species branches was only less than two (see Methods).

### A range of decay rates among ortholog families

After removing the residues with phylogenetic anomalies, the branch-specific substitutions, including the #1# branch, added up to 168,768 (96.3% of all) amino acid substitutions, and we relate the further analysis only to these residues. They were used to obtain relative branch lengths, overall branch-specific substitution rates, and when scaled to the branch length from timetree, that conform to a close range of 0.036–0.043 amino-acid substitutions per site per Mya (Table 1). Of course, given that the timetree branches were also calculated from molecular data, this rate consistency is not surprising *per se* (Hedges *et al.* 2006; Blair Hedges and Kumar 2009; Kumar *et al.* 2017). Still, we confirm that the cumulative analysis of substitutions supports a clock-like divergence hypothesis. However, this does not exclude that a subset of proteins could show episodic rates, and we went on to examine this specifically.

**Figure 4:**
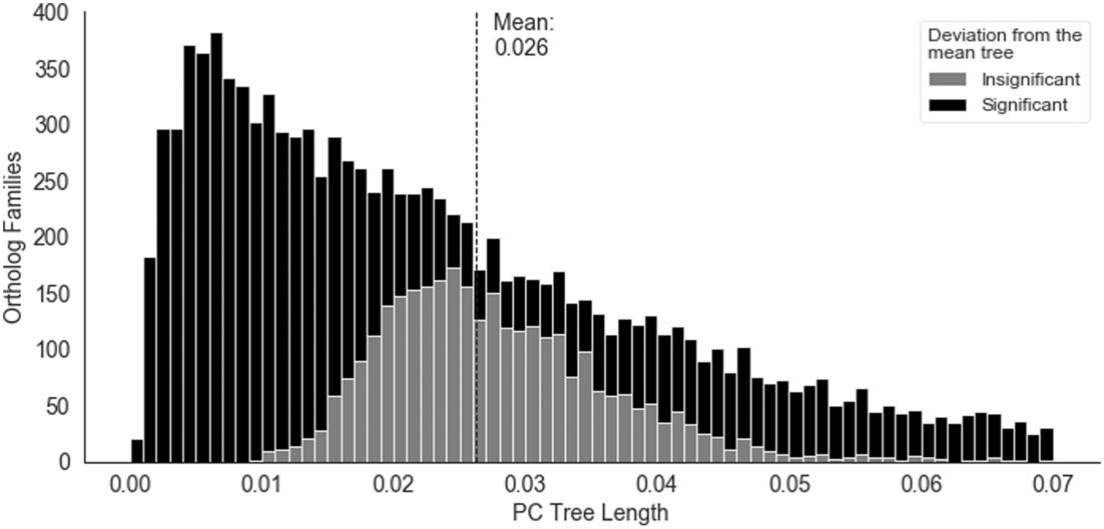
PC tree length distribution (stacked histogram), mean tree total length is marked by the dashed line. Shading represents the significance of departure from the mean tree based on the Z-statistic for the branch length aware RF score (Robinson and Foulds 1979).

For this, we calculated Poisson corrected branch lengths for every ortholog family with at least one substitution (1053 families had no substitution—see Supplementary Figure S1). These were then compared to the average branch length across all families, which we call the “mean tree” (Table 1). We then used the branch length aware RF metric to compare all constituent gene family tree lengths with the mean tree length and used a *Z*-test for testing significant differences (Robinson and Foulds 1979) (details in Methods). We found that 73% of substituted families showed a significant departure from the average tree, testifying to the expectation that each ortholog family can have its own rate. Hence, the overall rates depicted in Table 1 constitute a mixture of family-specific rates. The tree length distribution is plotted in Figure 4, showing a bias toward shorter trees. This is in line with the expectation that protein evolution is generally constrained by negative selection.

### Lineage-specific rate fluctuations

The RF score does not distinguish between an average rate difference versus strong branch-specific deviations. To detect significant deviations in the lineage-specific divergence rate of proteins, we needed to take a different approach. Here, we wanted to test that on the given tree for any ortholog family with “N” substitution events, the events are distributed along the branches as per expectation. Hence, to derive the expected distribution of events on each branch we performed 100,000 simulations for each value of *N* substitution events observed among the ortholog families (Supplementary Figure S2, see methods). The relative lengths of each branch were passed as the probability of an event occurring on the given branch (Table 1). The resulting distribution of substitution events on every branch was used to calculate the corresponding two-tailed *P*-value for obtaining the observed number of branch-specific substitutions. Finally, to detect significant deviations from the expectation, we performed Bonferroni correction on the five branches being tested in each ortholog family.

We found 608 orthologous proteins that were either slow- or fast-evolving on at least one branch (Table 2). Five hundred and thirty proteins were fast-evolving on just one branch, 160 were slow-evolving on just one branch, and only six proteins showed significant departure from the expected rate in the same direction on more than one branch. Conversely, 87 proteins showed significant departures in opposite directions on different branches, and among them, 63 were slow-evolving only on the gibbon branch and fast-evolving on one of the other branches. We observe that the slow-evolving proteins were enriched on the gibbon branch; this is most likely a result of reduced statistical power to detect less than expected substitutions on the other branches.

**Table 2:** Branches with different than expected substitutions

Branches	Ortholog families	
	Lower than expected	Higher than expected
Human	17	92
Chimpanzee	8	101
#1#	2	79
Gorilla	15	137
Gibbon	117	121
Human, Chimpanzee	—	1
Gorilla, #1#	—	1
Gorilla, Gibbon	2	—
Gorilla, #1#, Human	1	—
Gorilla, #1#, Human	1	—

### Genes evolving at a slower than expected rate

Among the 117 proteins with a lower-than-expected substitution on the gibbon branch, 54 did not have higher-than-expected substitution on any other branch (Supplementary Table S1). For example, NELL2 (neural EGFL like 2) protein had no substitution in the gibbon lineage over 826 aligned residues but had one, three, and four substitutions in human, chimpanzee, and gorilla lineage, respectively (Supplementary Figure S3). The remaining 63 proteins, with lower-than-expected substitution on the gibbon branch, had higher-than-expected substitutions on one of the other branches, including four proteins that were fast-evolving on the otherwise short #1# branch. BMP8B (bone morphogenetic protein 8b), with a 402 residue alignment, had five substitutions on the gorilla branch and three substitutions on the #1# branch but only one substitution on the gibbon branch (Supplementary Figure S4). NDUFAF6 (NADH: ubiquinone oxidoreductase complex assembly factor 6) also had a complex evolutionary trajectory; while all 333 residues of NDUFAF6 were retained in the final alignment, yet, the gibbon lineage had only one substitution while the #1# branch had five substitutions (Supplementary Figure S5).

### The fastest evolving proteins

One of the initial goals of our investigation was to identify proteins that diverge rapidly in particular lineages since they may be indicative of a functional change that leads to an evolutionary novelty over time (Domazet-Loso *et al.* 2007). Genes with a significantly higher than expected number of substitutions are listed in Supplementary Tables S5–S9. The lineage-specific fast-evolving proteins are distributed across all branches (Table 2). Even the #1# branch has 79 proteins with a higher-than-expected number of substitutions (Supplementary Table S9). KCNV2 was the only protein with a more than expected number of substitutions on both human and chimpanzee branches, but it was not fast diverging on the ancestral #1# branch (Table 3).

**Table 3:** Genes with a complex evolutionary history

Ortholog family/human transcript	Gene	Branch-specific substitutions					Align overlap	Align sat
		Human	Chimp	Gorilla	Gibbon	#1#		
ENST00000396124	NDUFAF6	0	1	2	1	5	333	100
ENST00000382082	KCNV2	12	12	0	3	2	545	100
ENST00000359741	SLC39A14	2	0	1	4	17	485	99
ENST00000274520	IL9	0	0	0	6	4	144	100

We manually curated a list of the five most divergent proteins on each branch (Table 4). Three human genes, ADCYAP1, PSORS1C2, and BTNL2, are associated with neuronal phenotypes, such as schizophrenia (Supplementary Table S10) (Hashimoto *et al.* 2007; Gusev *et al.* 2019; Amare *et al.* 2020). The most closely related paralogs of these genes were traced to the common ancestor of jawed vertebrates. Thus, the genes appear to be fast diverging even in the absence of recent duplication (Yates *et al.* 2020). This also stands true for the top genes on the other branches. Calmuenin, RNF128, SLC39A14, and S100Z were the most divergent genes on the chimpanzee, gorilla, #1#, and gibbon branch, respectively. Also, IL9, the second-fastest diverging protein on the #1# branch, did not have a single human-, chimpanzee-, or gorilla-specific substitution (Table 3), indicating that the rapid divergence on the ancestral branch was followed by absolute conservation along both descendent lineages.

**Table 4:** Genes diverging rapidly on a particular branch

Branch	Ortholog family/human transcript	Gene	Branch-specific % subs per site	Align overlap	Align Sat	Most recent duplication in the last common ancestors of
Human	ENST00000450565	ADCYAP1	7.60	171	97.16	Jawed vertebrates
Human	ENST00000637878	PVALEF	3.76	133	99.25	—
Human	ENST00000008938	PGLYRP1	3.57	196	100.00	Bilateral animals
Human	ENST00000259845	PSORS1C2	2.94	136	100.00	—
Human	ENST00000454136	BTNL2	2.88	243	89.67	Jawed vertebrate
Chimpanzee	ENST00000542996	CALU	6.25	320	99.07	Bilateral animals
Chimpanzee	ENST00000296280	MASP1	6.18	518	74.11	Jawed vertebrates
Chimpanzee	ENST00000380041	SCML1	5.21	326	99.09	Bilateral animals
Chimpanzee	ENST00000342995	CXorf67	3.11	386	97.97	Chimpanzee
Chimpanzee	ENST00000343470	LYAR	2.65	378	100	—
#1#	ENST00000359741	SLC39A14	3.51	485	98.98	Bilateral animals
#1#	ENST00000274520	IL9	2.78	144	100.00	—
#1#	ENST00000299191	C16orf78/EZHIP	2.27	264	100.00	—
#1#	ENST00000292894	THAP8	2.16	231	100	Bilateral animals
#1#	ENST00000625099	SLC22A18AS	1.99	251	99.2	—
Gorilla	ENST00000255499	RNF128	7.26	317	78.86	Bilateral animals
Gorilla	ENST00000254976	SNAP25	4.37	206	100.00	Vertebrates
Gorilla	ENST00000651546	CARD8	3.67	354	87.19	—
Gorilla	ENST00000613760	WDR38	3.30	303	98.70	Animals and Fungi
Gorilla	ENST00000255977	MKRN1	3.17	473	98.13	Simians
Gibbon	ENST00000513010	S100Z	15.22	92	93.88	Vertebrates
Gibbon	ENST00000345088	DPPA3	14.97	147	92.45	Placental mammals
Gibbon	ENST00000393330	TSPAN8	12.66	237	100.00	Bilateral animals
Gibbon	ENST00000397301	TNNT3	12.57	167	97.09	Bilateral animals
Gibbon	ENST00000523047	SMIM23	12.14	140	96.55	—

**Figure 5:**
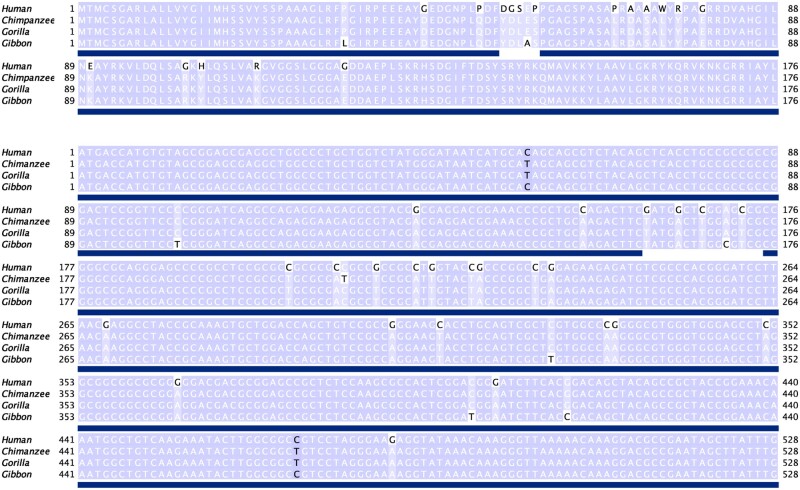
Protein and CDS multiple sequence alignments of the ADCYAP1 orthologs. Sites retained in the final alignment are underlined by the blue blocks.

ADCYAP1 is the most divergent human gene in our analysis. A previous study has shown that the gene went through accelerated adaptive evolution (Wang *et al.* 2005). However, in the absence of genome sequences from other species, they did not compare its evolutionary rate with other genes. The gene encodes a neuropeptide: Pituitary adenylate cyclase-activating polypeptide (PACAP). PACAP, along with its receptor PAC1 (ADCYAP1R1), plays a crucial role in regulating fear physiology and stress response (Ressler *et al.* 2011). PACAP is known to stimulate adenylate cyclase in pituitary cells and promote neuron projection (Emery *et al.* 2013). ADCYAP1 has biased expression in appendix, brain, gall bladder, testis, and nine other tissues (Fagerberg *et al.* 2014). Within the sites retained in the final alignment, there were 13 substitutions along the human branch and one substitution on the gibbon branch; at the nucleotide level, there were 20 human-specific and seven other substitutions within the same region (Figure 5). It is important to note here that all human-specific substitutions are A/T to G/C, implicating biased gene conversion as a possible mutational mechanism (Berglund *et al.* 2009; Galtier *et al.* 2009). A comparison of both amino acid and nucleotide alignment revealed that the five amino acid residues stretch not included in the final alignment included only five nucleotide substitutions. However, they resulted in five contiguous substitutions at the protein level, causing these residues’ exclusion from the final alignment. This stretch had four human-specific amino acid residues emanating from four nucleotide substitutions, and including these five residues raised the human-specific % substitutions per site to 9.7.

Inflation of the estimated % substitutions per site was also observed among the fastest evolving proteins on the other branches. Calmuenin (CALU) the most divergent chimpanzee protein had three substitute residues that were filtered from the alignment (Supplementary Figure S6), including these substitution takes the chimpanzee-specific % substitutions per site to 7.1. CALU is a calcium-binding protein involved in protein folding and sorting in the endoplasmic reticulum (Philippe *et al.* 2017). SLC39A14, the most divergent protein at the #1# branch, had three substituted residues that were filtered (Supplementary Figure S7), including them takes its #1#-specific % substitutions per site to 4.1. SLC39A14 is a divalent metal transporter known to be associated with neurodegeneration and early-onset Parkinsonism-dystonia (Balint and Bhatia 2016). The fastest evolving protein in the gorilla lineage, RNF128, had two stretches of three substitute residues that were filtered from the final alignment and including these residues increased the gorilla-specific % substitutions per site to 9 (Supplementary Figure S8). RNF128 is a transmembrane zinc finger protein that functions as an E3 ubiquitin ligase in the endocytic pathway, and its expression limits IL2 and IL4 production by T lymphocytes (Anandasabapathy *et al.* 2003). S100Z, the fastest evolving protein on the gibbon lineage, does not have any filtered substituted residues and hence has a gibbon-specific 15.2% substitutions per site (Supplementary Figure S9). S100Z is a member of the S100 protein family and contains two calcium-binding EF-hands, and exhibits specific expression patterns (Gribenko *et al.* 2001).

It is noteworthy that all of these top genes are generally highly conserved genes, present throughout all multicellular life forms and all with solved protein structures. Hence, they would not be classified as orphan genes.

## Discussion

Using the classic approach to study protein-sequence divergence rates across time, we find that hundreds of proteins depart from the null expectation of constant decay rate even in the rather shallow phylogeny of Hominoids. Given that the fluctuations include both higher divergence and higher conservation, it appears that these effects tend to cancel each other out in the aggregate data, leading to the emergence of an overall clock-like pattern. This interpretation reconciles the molecular clock departure patterns in individual protein families with the generally accepted notion that molecular data can be reliably used to derive splitting time estimates of taxa. On the other hand, this interpretation also supports models of episodic evolution. Given that each lineage showed a separate set of proteins with significant rate changes, one can project that increasingly larger fractions of proteins would have undergone episodic rate changes in deeper phylogenies, although this inference will still need to be rigorously tested.

### Technical considerations

The tempo of protein evolution through point mutation is measured by detecting divergent sites. However, accurate identification of such sites relies heavily on a proper alignment achieved by juxtaposing conserved sites. A precise alignment of ancestrally derived sites is vital for a comparative genomic analysis involving multiple species. Thus, to conduct a thorough investigation, we carefully created a curated dataset that goes beyond the reciprocal BLAST hit approach that is used to create ortholog databases across large phylogenetic distances (Kriventseva *et al.* 2019). We needed to identify true orthologs derived from the same ancestral gene of the extant species’ last common ancestor. Identification of definite orthologs, even among closely related species, is complicated by evolutionary processes such as deletion, duplication, and gene conversion. Our reliance on synteny to identify a set of collinear genes verified that these positional orthologs were homologous and situated at loci with conserved gene order.

Three findings validated our confidence in the chosen approach. First, most ortholog families did not show considerable variation in their protein lengths. Second, we obtained a high mean alignment saturation level even after removing all gaps. Third, there was an overwhelming abundance of identical sites within the aligned columns, and the substituted sites were heavily enriched with branch-specific substitutions. Another indication of our final alignments’ reliability was that all 52 hypermutable sites were in different families. Even a partial misalignment can easily lead to erroneous detection of multiple hypermutable sites. Hence, the lack of more than one hypermutable site in any family should be considered an additional testament to the alignment quality. Here, we posit that our rigorous approach, necessary to create a high-confidence set of ortholog families, provided an opportunity for comprehensive analysis of a large dataset. Furthermore, by not considering unequivocally alignable sets, our approach is actually conservative with respect to measuring substitution rate departures.

### Overall clock-like patterns

Before analyzing the individual gene families, we calibrated our dataset against a given scale. For this, we normalized our branch-specific substitution rates with their respective evolutionary time estimates from the time-tree (Kumar *et al.* 2017). A constant rate of sequence divergence for each protein family predicted by the molecular clock hypothesis should result in similar overall substitution rates on all branches. When scaled to the time-tree, which was itself estimated from a combination of paleontological and molecular data, our estimated branch-specific substitution rates confirmed that the substitutions per site per Mya falls within a close range on all branches, but it is known that some fluctuation exists in the hominid tree that leads to overall branch length changes (Moorjani *et al.* 2016; Mello and Schrago 2019). However, our focus was not on these overall effects but on the lineage-specific fluctuation at the family level. Comparison of the individual family trees with the mean tree by the branch-length aware Robinson–Foulds (RF) metric revealed that nearly three-fourth of substituted families showed statistically significant departure from the mean tree (Robinson and Foulds 1979). Moreover, upon testing the direction of departure from the mean tree, we found that two out of three families departing from the mean tree lengths were shallower than the mean tree. This suggests that even when each family has its own specific rate, protein evolution is largely constrained by negative selection.

### Lineage-specific effects in protein families

While differences in family-specific rates were expected, given the phylogenetic proximity of species under investigation and the stringency of our alignment protocol, the number of families with lineage-specific rate deviations in individual families were expected to be low. Yet, we found 608 families that showed significant lineage-specific deceleration or acceleration of rates. For the accelerated ones, we find roughly 13 families per million years of divergence over the entire tree. Hence, if one would analyze a much deeper phylogeny, *e.g.*, of a divergence time of 500 million years, one should conservatively expect several thousand families with phases of acceleration. If deceleration phases, which are harder to detect on the shallow branches, compensate for these acceleration phases, one could still end up with an overall clock pattern for most families, but departures could also be frequent. In fact, this possibility of episodic evolution was intensively studied early on, based on mathematical considerations and simulations (Hudson 1983; Gillespie 1984). Hudson (1983) suggested, “It is concluded that the constant-rate neutral model is highly improbable,” and Gillespie (1984) record, “… our statistical analysis suggests that the course of molecular evolution is episodic…”. Interestingly, while the databases were growing, this issue had not been systematically revisited so far. Our analysis here fully supports these statements.

While our analysis will still need to be confirmed in other datasets and ideally also in deeper phylogenies, it appears that the current evidence does not permit an unimpeachable assumption of a constant rate decay model for protein evolution as the null hypothesis, as recently proposed by Weisman *et al.* (2020). Their analysis was mostly guided by asking which fraction of proteins would decay with a sufficiently high rate over time to let it escape homology detection algorithms (such as BLAST). This is of particular relevance for identifying the most credible candidate genes for *de novo* evolution. They concluded that for a large number of genes one would not be able to distinguish *de novo* evolution from overall fast evolution when a rate calibrated from a shallow phylogeny is projected to a deeper phylogeny. However, if this shallow phylogeny included acceleration phases for the protein in question, it could yield an ambiguous conclusion for long-term evolution.

Although we have not studied changes in substitution rates in duplicated genes here, we posit that the duplication-divergence with subsequent constraints model suggested by Domazet-Loso and Tautz (2003) is supported by our findings, given that even orthologous proteins show clear signs of episodic evolution phases. If this model applies, it may cast doubt on candidates of *de novo* gene evolution that are identified by the method of Weisman *et al.* (2020), since this method cannot trace the fast phase of evolution after the duplication of genes, given that it relies on rate estimates derived from the more constrained history of evolution. Hence, it remains very challenging to prove *de novo* evolution of genes in deep phylogenies, even when including synteny considerations (Vakirlis *et al.* 2020b). On the other hand, several examples have now indubitably identified *de novo* evolved functional genes in shallow phylogenies (Heinen *et al.* 2009; Prabh and Rödelsperger 2019; Xie *et al.* 2019; Vakirlis *et al.* 2020a), that allow us to trace the exact evolutionary history of the new genes and thus validates that evolution of functional new genes out of noncoding sequences is possible.

### Extreme genes

Genes with extreme changes in substitutions rates are candidates for having a specific adaptive relevance for the respective taxon in whose branch they occur. In this analysis, we focused on each branch’s five fastest diverging genes, but we emphasize that the list could easily be extended (Supplementary Tables S5–S9). Among the 25 highly divergent genes, five at every branch, only one was recently duplicated, confirming that rapid sequence divergence can occur even in the absence of duplication. In humans, we found that the rapidly evolving genes are involved in essential biological processes such as cognition. These genes were associated with disease phenotypes such as schizophrenia, autism, and blood pressure. We identified ADCYAP1 as the most divergent human protein-coding gene. It encodes a 176 amino acid residue protein that contains 17 human-specific substitutions, which estimates to a substitution frequency of 10%. The high substitution rate could have been fostered by a biased gene conversion process, as all nucleotide substitutions in humans were A/T to G/C. Despite the high divergence, PACAP, the product of ADCYAP1, remains a key mediator of fear physiology and stress response in humans and mice. This gene’s biological relevance, coupled with the lack of recent duplicates, affirms that the accelerated divergence did not result from functional redundancy. To our knowledge, no other protein has been shown to have such a high rate of human-specific divergence. It is necessary to emphasize that each lineage includes such lineage-specific highly accelerated genes, *i.e.*, it is not special for humans to find such cases, but it is a general pattern that accompanies species formation.

## Conclusion

Our analysis reveals a dynamic history of substitution rate changes in hundreds of protein families over a rather short evolutionary interval. The data suggest also that in the long-term evolution of proteins, the episodic acceleration and deceleration can potentially cancel each other out in the aggregated data. While this could give the impression of a long-term constant rate, which is often assumed as a null model for protein evolution, the actual history of the evolution of a given protein sequence is better described by episodic substitution models.

## Data availability

The data underlying this article are available in the article and its online supplementary material at https://github.com/neelduti/EpisodicEvolution2.
